# First person – Roger Smith and Igor Odintsov

**DOI:** 10.1242/dmm.049407

**Published:** 2022-01-27

## Abstract

First Person is a series of interviews with the first authors of a selection of papers published in Disease Models & Mechanisms, helping early-career researchers promote themselves alongside their papers. Roger Smith and Igor Odintsov are co-first authors on ‘ Novel patient-derived models of desmoplastic small round cell tumor confirm a targetable dependency on ERBB signaling’, published in DMM. Roger completed the research described in this article while a research technician in the lab of Marc Ladanyi at Memorial Sloan Kettering Cancer Center, New York, NY, USA. He is now an MD/PhD student in the lab of Marc Mendillo at Northwestern University, Chicago, IL, USA, investigating stress response pathways in cancer. Igor completed the research described in this article while a postdoctoral fellow in the lab of Marc Ladanyi at Memorial Sloan Kettering Cancer Center. He is now a resident anatomic pathologist at Brigham and Women's Hospital, Boston, MA, USA.


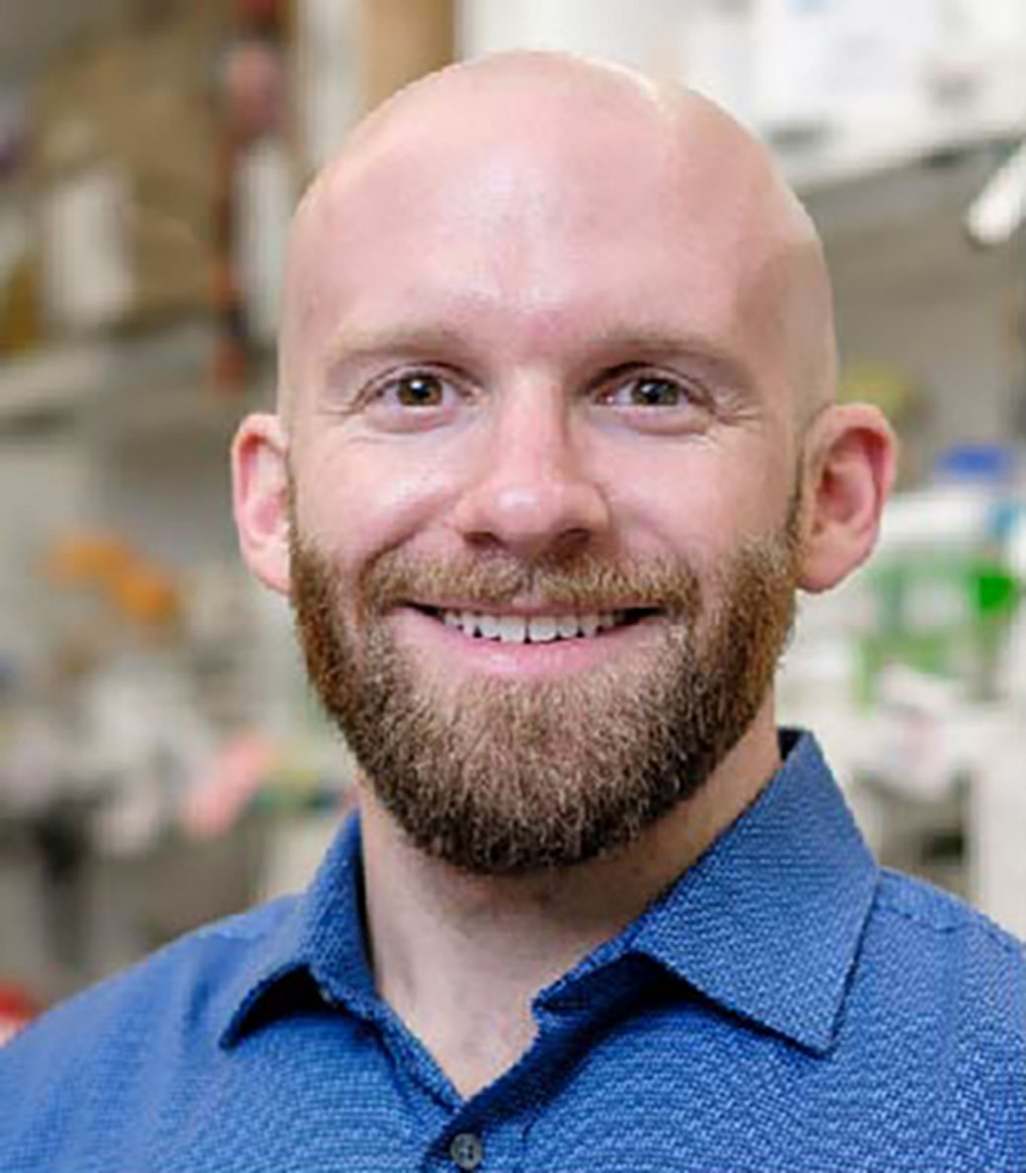


**Roger Smith**

**How would you explain the main findings of your paper to non-scientific family and friends?**

Desmoplastic small round cell tumor (DSRCT) is an aggressive cancer that affects most often male pediatric and adolescent patients. The disease is caused by a specific genetic alteration that fuses two genes to create a potent fusion oncogene that drives DSRCT. The overall survival is 15% at 5 years. A lack of disease models to study the biology of the disease and develop therapy has contributed to the poor understanding of this cancer. In our study, we developed and/or characterized four new DSRCT cell lines (only one existed prior to the start of our study) and one patient-derived xenograft model, which means a mouse model of a human tumor sample. Comparative analysis of large-scale gene expression data of five sarcomas demonstrated that the epidermal growth factor receptor (EGFR) family pathway was more activated in DSRCT tumors compared to other sarcomas. Using our new models, we were able to validate EGFR as a significant contributor to growth of these tumors and demonstrate that targeting EGFR with drugs that are currently approved by the U.S. Food and Drug Administration (FDA) for other cancers can reduce growth of multiple DSRCT disease models.

**What are the potential implications of these results for your field of research?**

The disease models we describe provide new tools to understand the biology of DSRCT. This is underscored by the number of requests that we have received from researchers from around the world for these novel DSRCT cell lines. A deeper understanding of the cellular mechanisms that drive growth of this tumor will likely reveal new targets that can be exploited for therapy. Our finding that available FDA-approved drugs targeting the EGFR pathway can slow growth of the DSRCT disease models opens the possibility that patients may benefit from these drugs as part of their therapy, especially when all currently used agents fail.

**What are the main advantages and drawbacks of the model system you have used as it relates to the disease you are investigating?**

Our cell lines can be grown in cell culture or as xenograft tumors in immunocompromised animals. This allows for flexibility with available expertise in different laboratories. The xenograft tumor models will be useful in developing and testing new forms of therapy. One drawback is that not all our cell lines grow as subcutaneous xenografts, which is the easiest way to assess *in vivo* growth. Another limitation is that we were unable to compare all the disease models to the original patient tumor that was used to develop the models and therefore cannot directly assess the fidelity of our models compared to some of the individual patient tumors.

“[…] a single agent targeting EGFR can slow growth of DSRCT xenograft models.”

**What has surprised you the most while conducting your research?**

The low success rate of generating DSRCT cell lines was very surprising, given the aggressive nature of this disease. At the time of submission of the manuscript, we successfully generated four cell lines from 55 different tumors (i.e. 7% success rate, compared to >50% for most other tumors we have attempted). Another surprise was the finding that a single agent targeting EGFR can slow growth of DSRCT xenograft models. Currently, DSRCT patients are treated with an intensive regimen of multiple chemotherapy drugs, radiation therapy and surgery. We hope that the addition of anti-EGFR therapy to the war chest to fight this disease will provide benefits to these young patients.


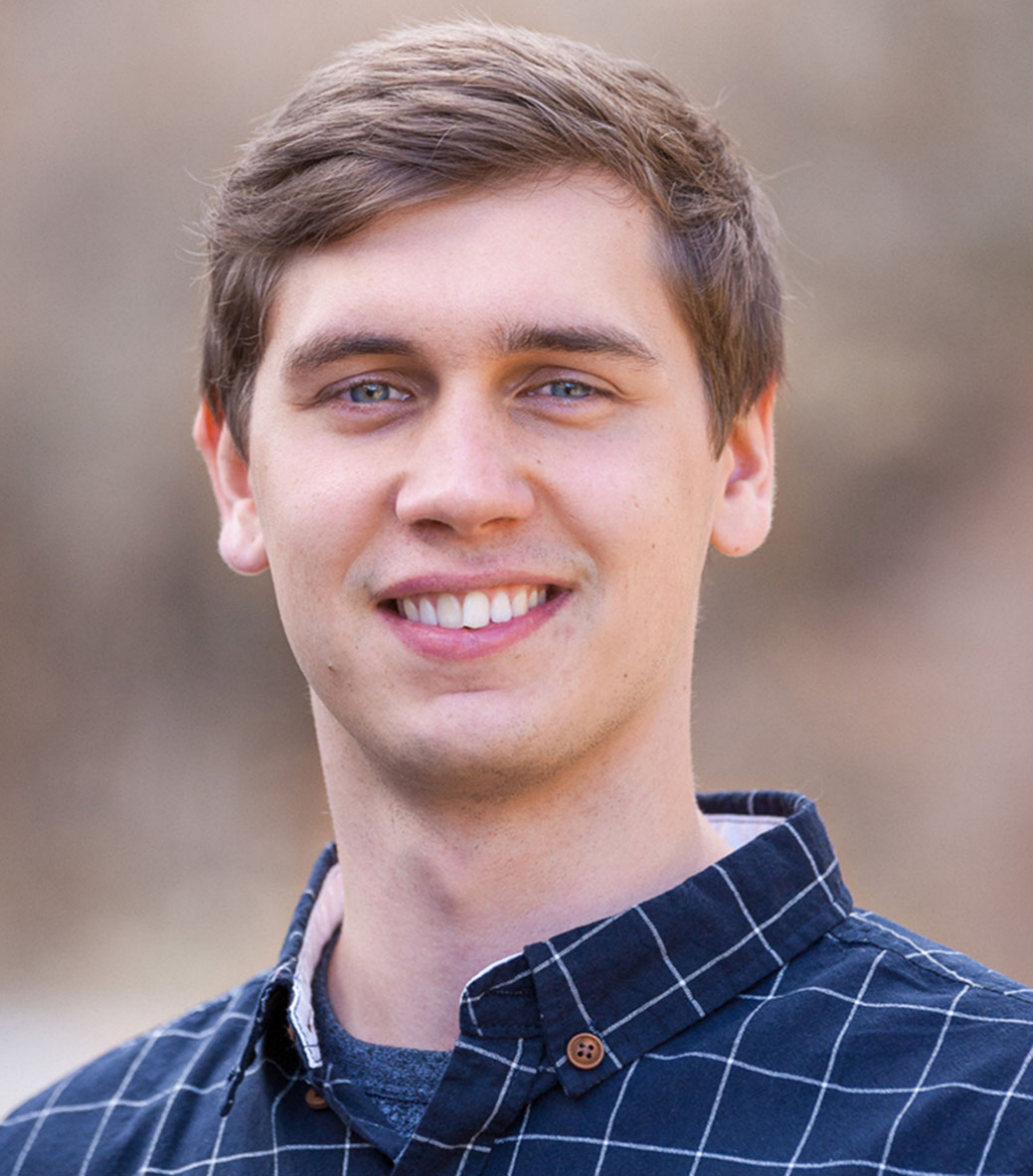


**Igor Odintsov**

**Figure:**
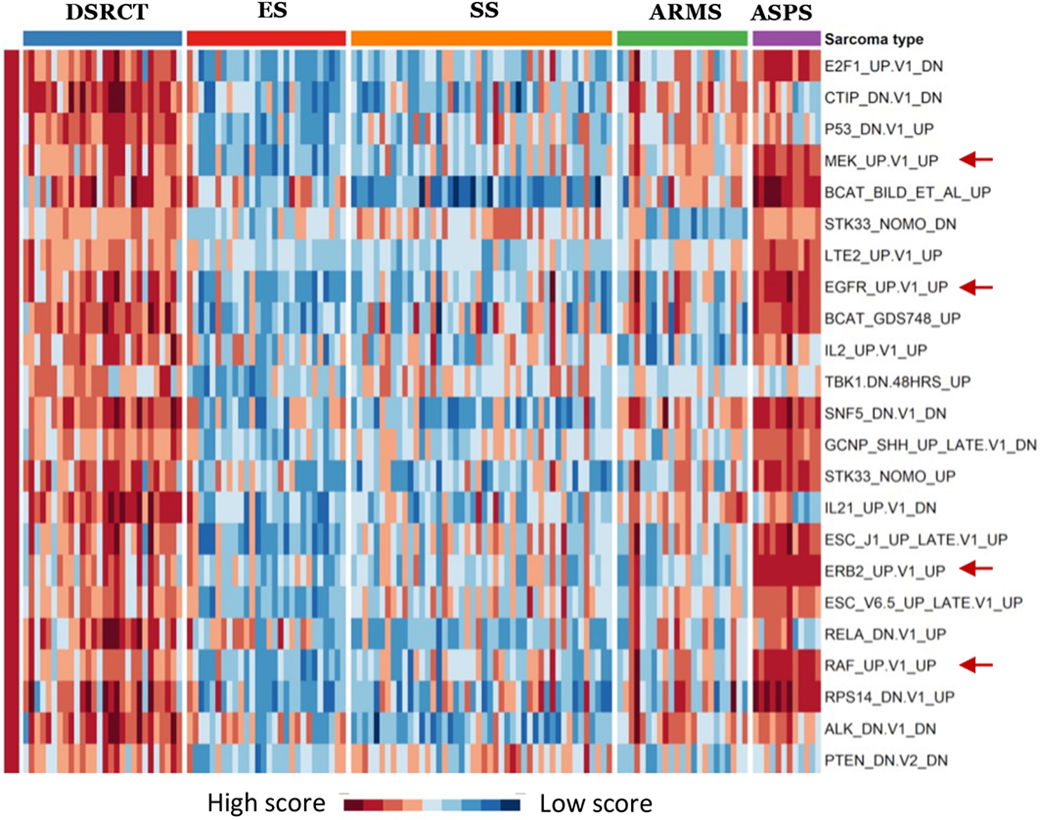
**Heatmap of differently enriched oncogenic signatures in five sarcomas**. Comparison of gene expression signatures show that the *EGFR* pathway is activated preferentially in DSRCT tumors compared to the other sarcomas.

**Describe what you think is the most significant challenge impacting your research at this time and how will this be addressed over the next 10 years?**

DSRCT is a very rare disease, which limits the opportunities to develop patient-derived tools such as cell lines and mouse models that allow us to better understand its unique biology. Therefore, obtaining the necessary preclinical data with many patient-derived preclinical models to support randomized clinical trials remains a major challenge. Our present study begins to address this problem with the successful generation and characterization of novel patient-derived models. To further expand our research tools with a complementary approach, we leveraged CRISPR/Cas9 genome-engineering strategies to generate isogenic models with gene fusions. Isogenic models have some advantages because there is less genetic variation than patient-derived models, and we have both had success with this approach for generating models of other fusion-driven cancers. We will continue to build on these two complementary strategies to better understand the fundamental biological pathways driving DSRCT pathogenesis and identify additional therapeutic targets. Moreover, sharing these models with the scientific community will foster more collaborations and accelerate progress to overcome this challenging disease.

“It is important for early-career scientists to feel fully engaged in the scientific research community […]”

**What changes do you think could improve the professional lives of early-career scientists?**

**RS:** It is important for early-career scientists to feel fully engaged in the scientific research community, especially with their peers. I was lucky to attend a Gordon Research Conference before the 2020 pandemic that included scientific and networking sessions specifically for graduate and postdoctoral trainees. This time was an excellent way to make new friends at the conference and new scientific colleagues from around the world. It would be great if more conferences adopted a similar component. Funding to support the ideas of young scientists is always important, and additional support to foster this community building can amplify scientific investments through collaboration.

**IO:** For early-career scientists, it is important to fully understand their potential career prospects. It is why, I think, proper mentorship should include individually tailored advising on career options. The versatility of scientific training allows for a wide variety of careers, and it is pertinent for an early-career scientist to appreciate it. Speaking from a personal example, after a thorough discussion with my mentor, I decided to continue my training as a pathologist by entering a residency program, to ultimately pursue a career as a physician-scientist.

**What's next for you?**

**RS:** I'm currently completing my PhD within the next year from the laboratory of Marc Mendillo, PhD, where I study how cancer cells co-opt protein homeostasis mechanisms to support their rapid proliferation and stress resilience. I will then return to the final 2 years of my medical degree training before completing residency and fellowship in a specialty to be determined. I am eager to gain clinical experiences that inspire questions for basic science investigation and, ultimately, translate results of these studies into improved treatment and management of human disease.

**IO:** I recently started pathology residency at Brigham and Women's Hospital in Boston, where I am pursuing a 3-year training in anatomic pathology. I am interested in soft-tissue pathology, and rare soft-tissue sarcomas driven by chimeric transcription factor oncoproteins, such as DSRCT, is one of my principal interests in the area. Brigham and Women's Hospital, in collaboration with Dana Farber Cancer Institute, accumulates a large number of patients with rare sarcomas and is uniquely suited for this purpose. I hope to return to academic research after my clinical training, to eventually become a physician-scientist.
